# Supplementary material for: A Novel Protein–Protein Interaction between RSK3 and IκBα and a New Binding Inhibitor That Suppresses Breast Cancer Tumorigenesis
Source: Cancers (Basel). 2021 Jun 14;13(12):2973. doi: 10.3390/cancers13122973 (PMC8231827; doi:10.3390/cancers13122973)
Supplement: Supplementary file 1 [file cancers-13-02973-s001.zip › cancers-1186035-supplementary.pdf]

cancers

MDPI

Supplementary Material

A Novel Protein–Protein Interaction between RSK3 and IκBα and a New Binding Inhibitor That Suppresses Breast Cancer Tumorigenesis

Hee-Sub Yoon, Sung Hoon Choi, Jung-Hyun Park, Jin-Young Min, Ju-Yong Hyon, Yeji Yang, Sejin Jung, Jae-Young Kim, Nam Doo Kim, Ji Hoon Lee, Eun Hee Han, Sung-Gil Chi and Young-Ho Chung

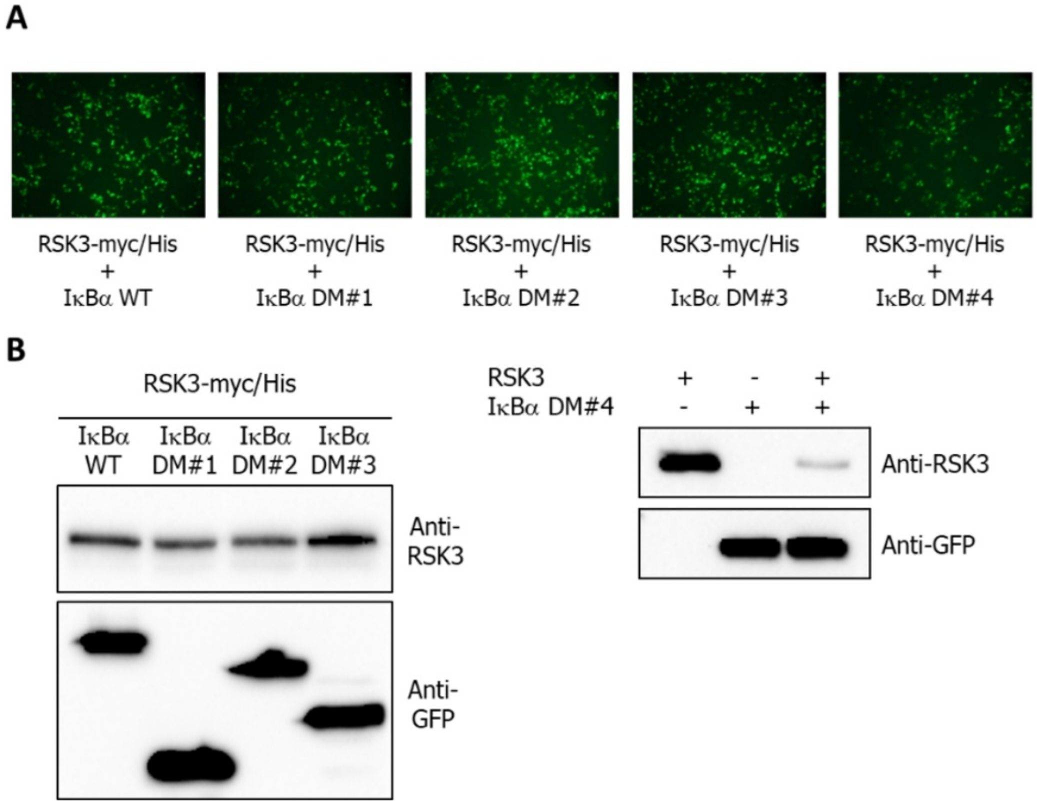

Figure S1. Expression of RSK3 and IκBα deletion mutant GFP fluorescence using ZOE Fluorescent Cell Imager and western blot. (**A**) Co-transfected with RSK3-Myc/His and GFP tagged IκBα WT or deletion mutant into HEK293 cell lines, and GFP expression was confirmed via ZOE Fluorescent Cell Imager. (**B**) Protein expression of RSK3-Myc/His and GFP tagged IκBα WT or deletion mutant was confirmed the using anti-GFP and anti-RSK3 antibody by Western blotting.

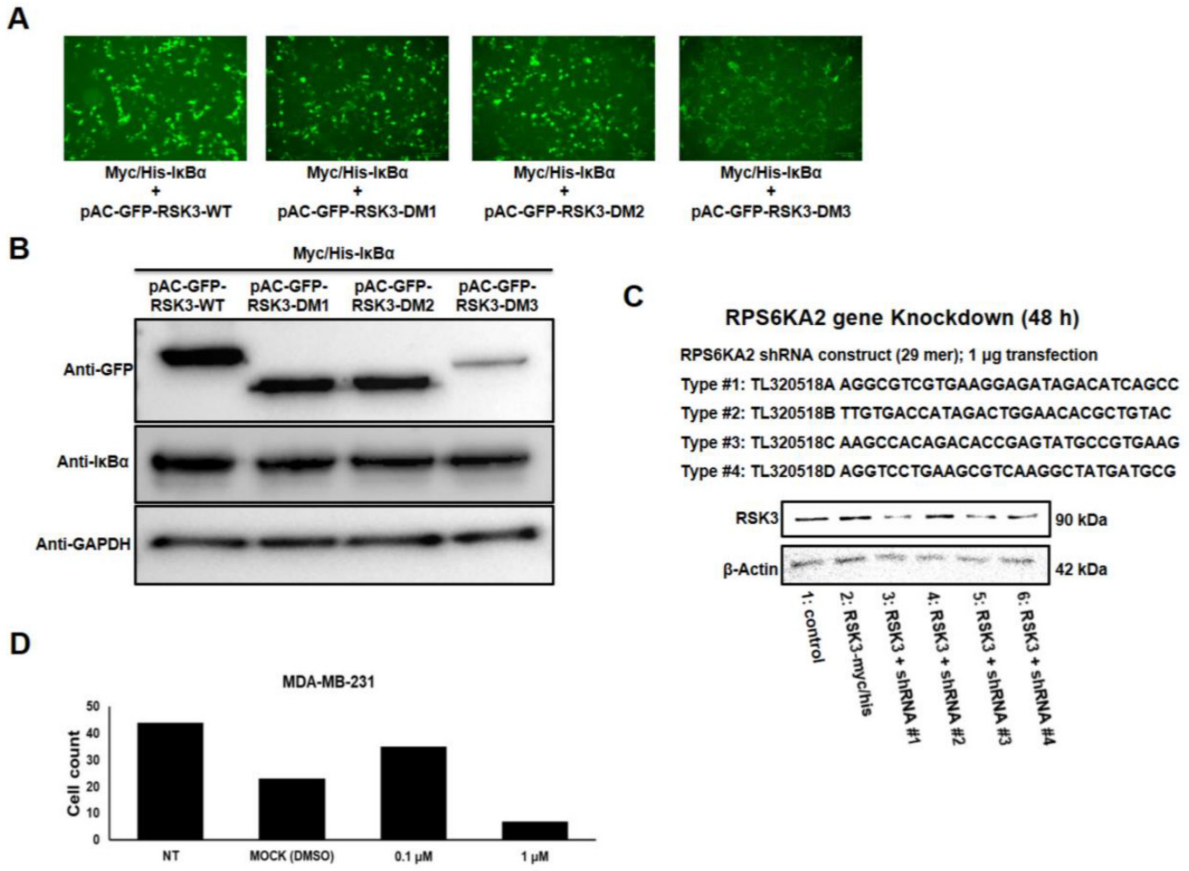

Figure S2. Expression of IκBα and RSK3 deletion mutant via ZOE Fluorescent Cell Imager and western blot, shRNA sequence of RSK3 and Foci assay cell count. (**A**) Co-transfection of Myc/His-IκBα with GFP tagged RSK3 WT and deletion mutant into HEK293 cells, and expression of GFP was confirmed via ZOE Fluorescent Cell Imager. (**B**) The western blot was performed that transfected and overexpressed of the Myc/His-IκBα and GFP tagged RSK3 WT and deletion mutant using anti-GFP and anti-IκBα antibody by Western blotting. (**C**) shRNA of RSK3 sequence and expression was confirmed the western blot using anti-RSK3 and anti-β-actin antibody. (**D**) Foci assay cell count grape. Cell was counted using ImageJ program.

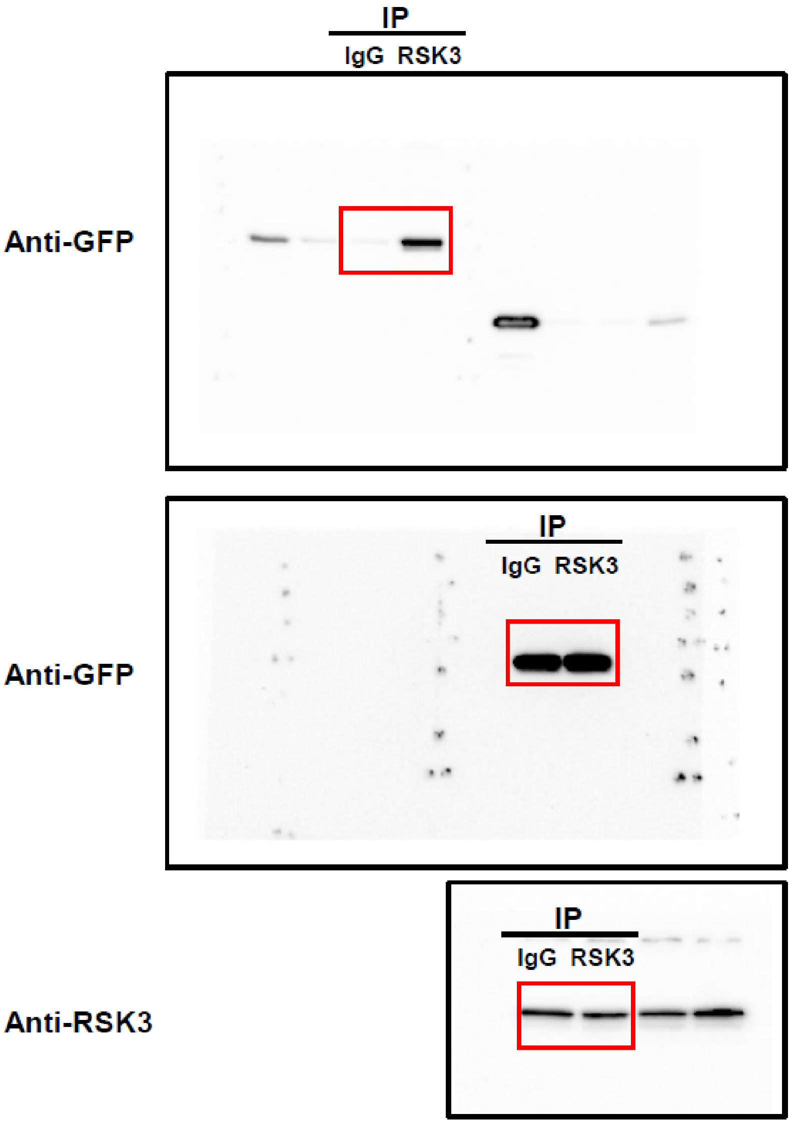

Figure S3. Uncropped Figure 1B.

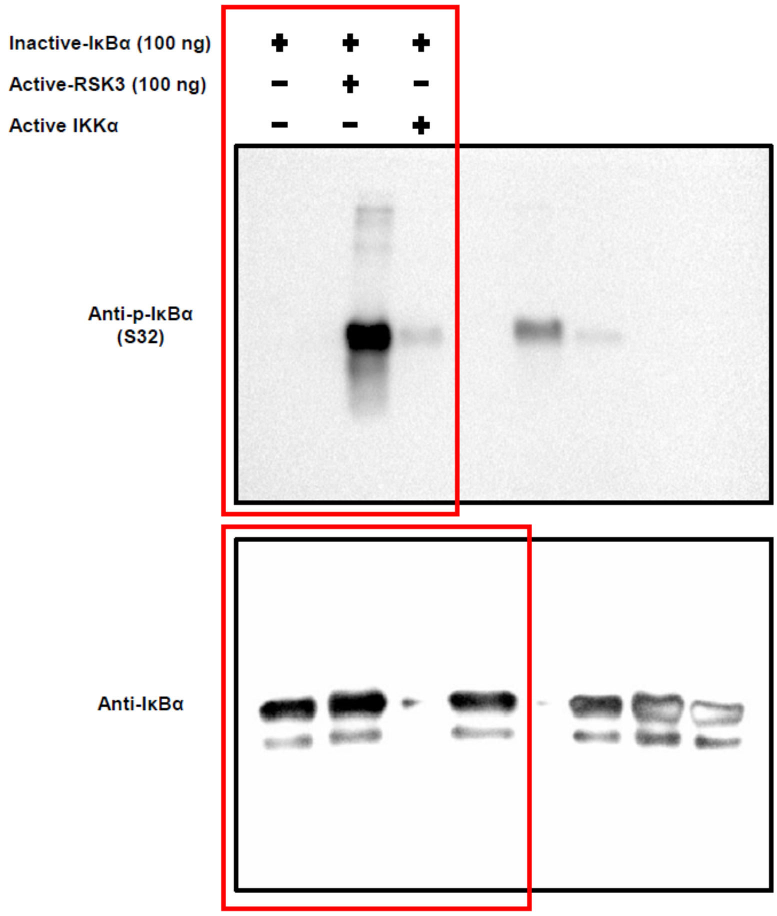

Figure S4. Uncropped Figure 1D.

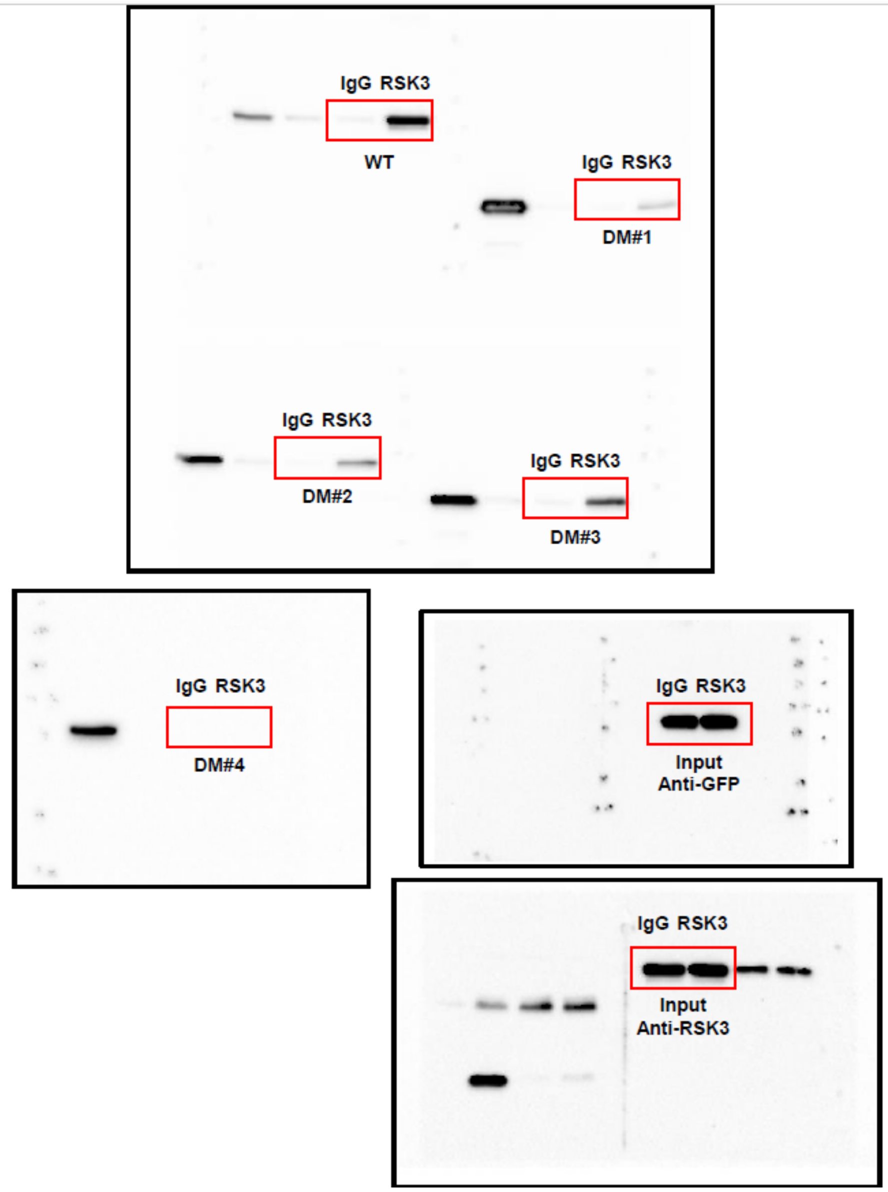

Figure S5. Uncropped Figure 2B.

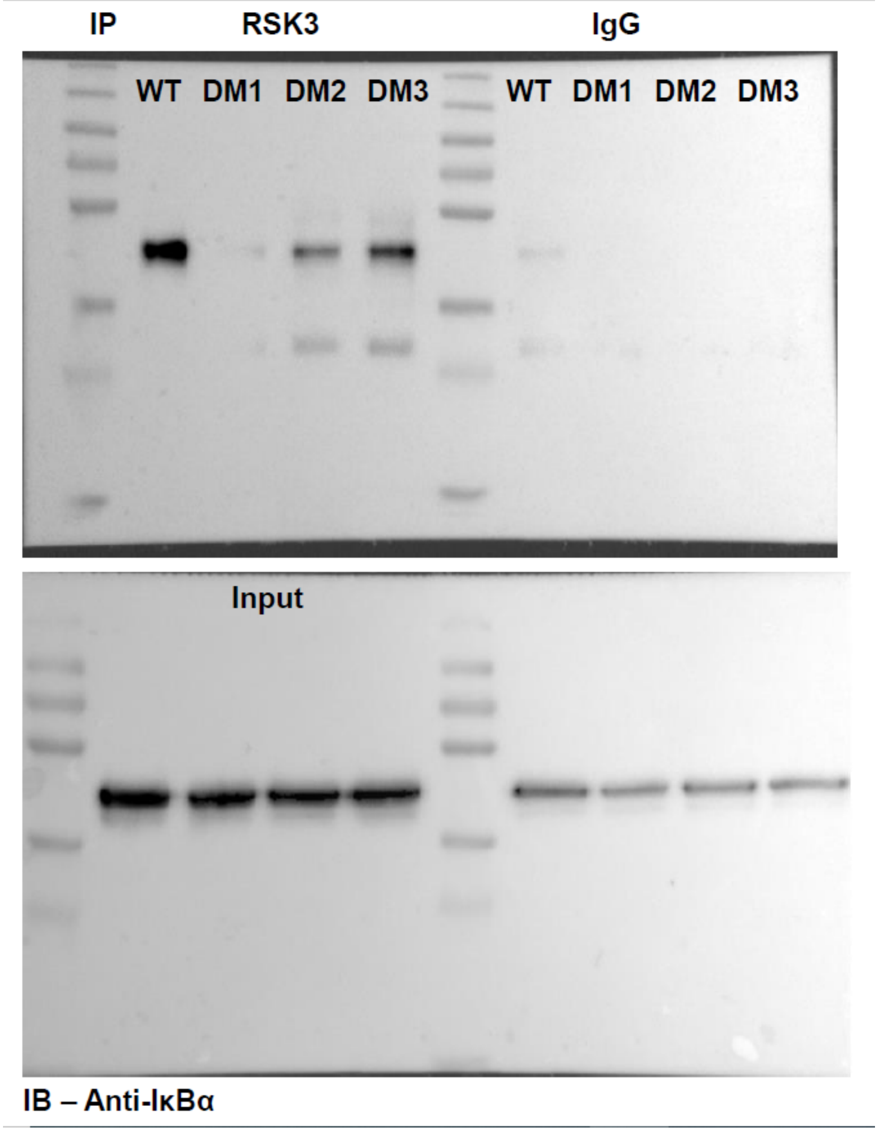

Figure S6. Uncropped Figure 2D.

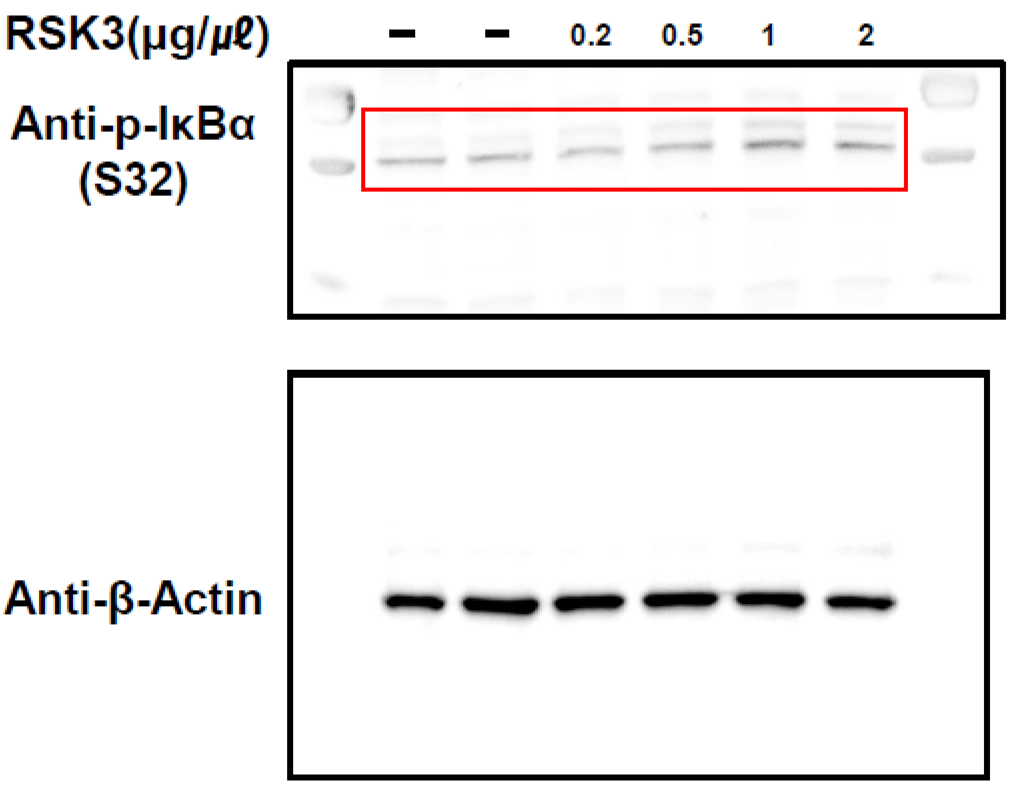

Figure S7. Uncropped Figure 3A.

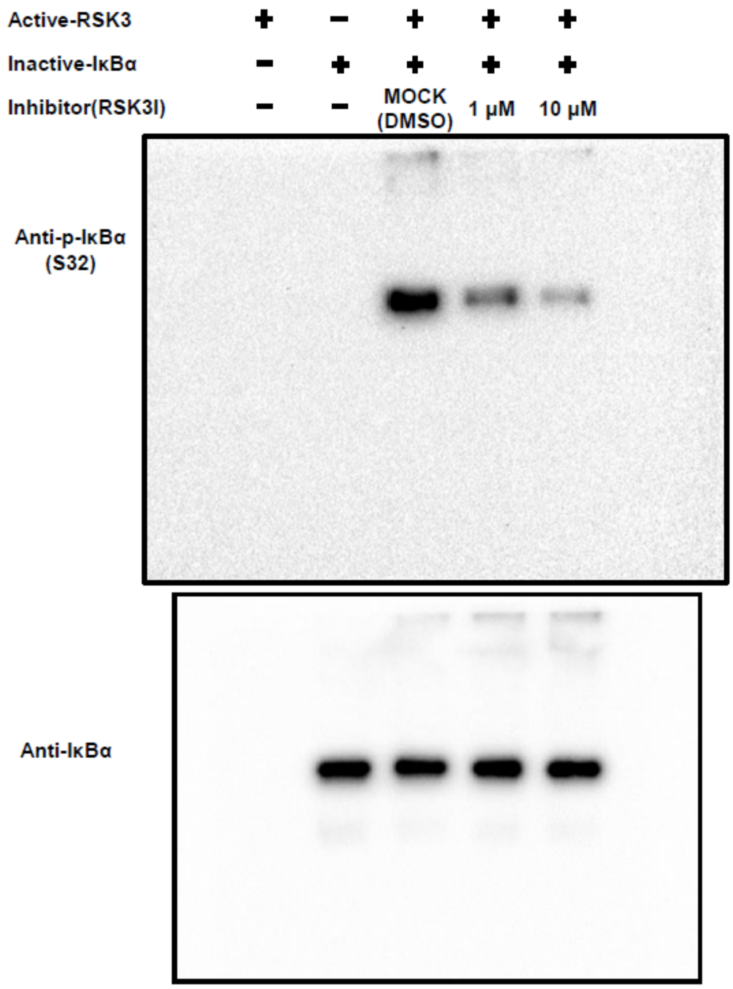

Figure S8. Uncropped Figure 4D.

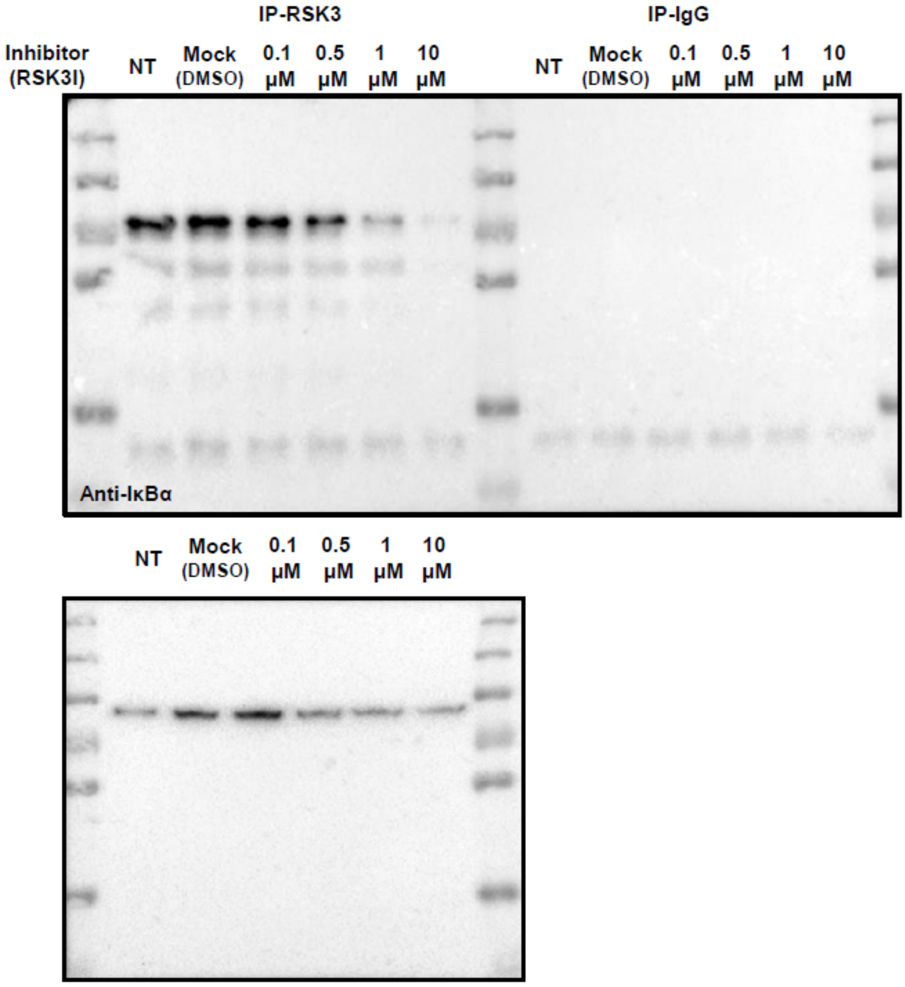

Figure S9. Uncropped Figure 4E.

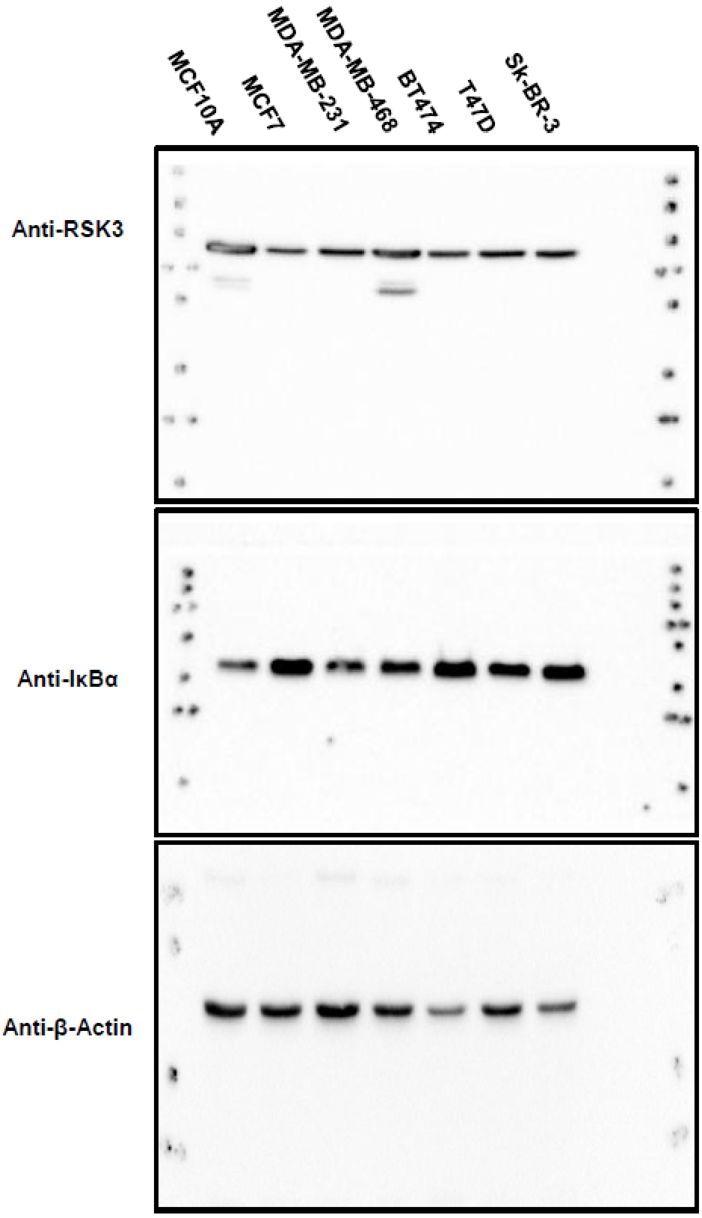

Figure S10. Uncropped Figure 5A.

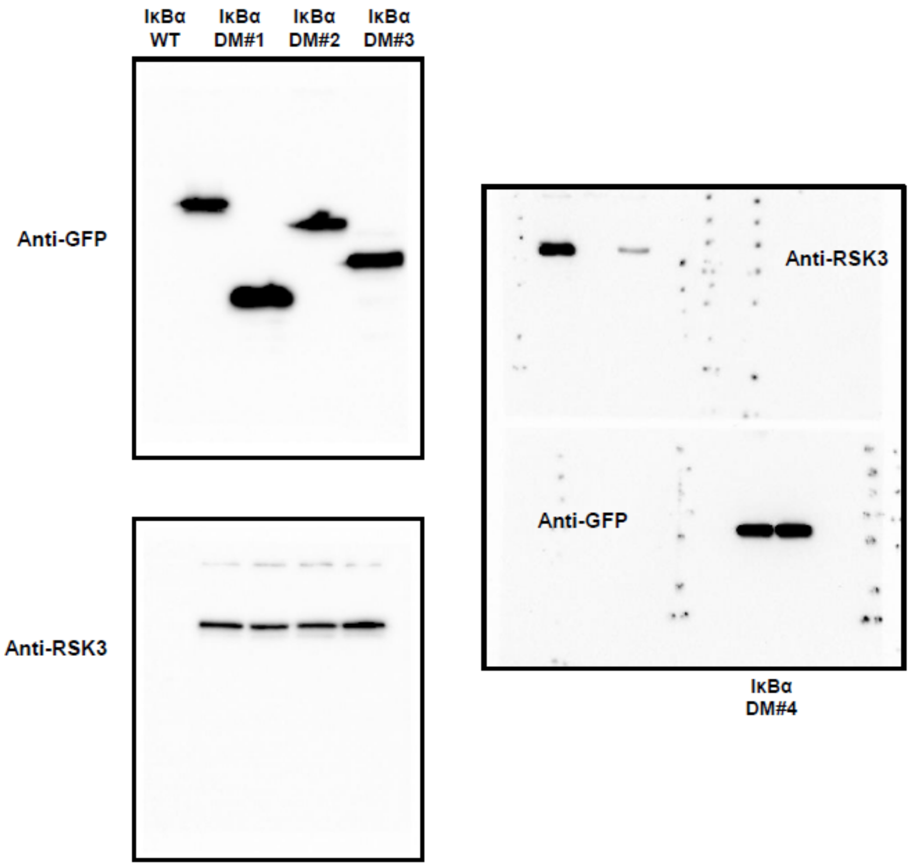

Figure S11. Uncropped Figure S1B.

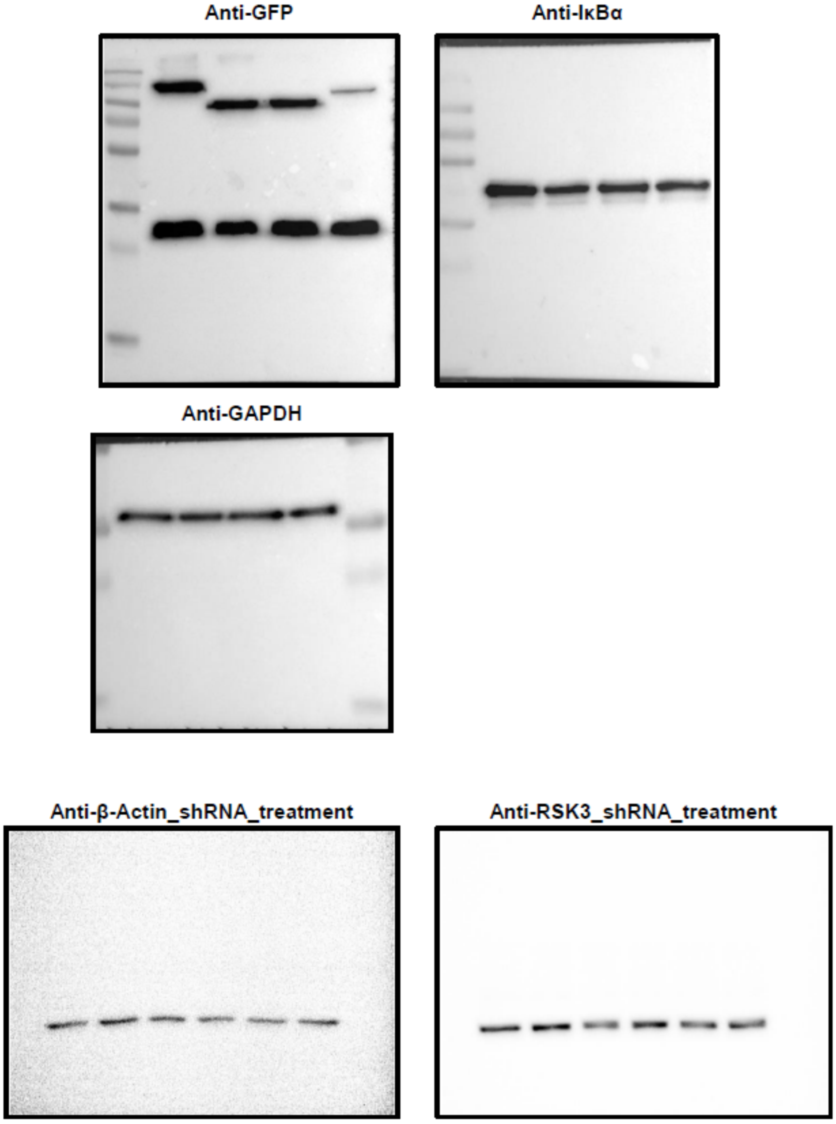

Figure S12. Uncropped Figure S2B.
